# Service user groups as co-applicants on a platform study for a trial

**DOI:** 10.1186/1745-6215-14-S1-O35

**Published:** 2013-11-29

**Authors:** Heather Morgan, Pat Hoddinott, Gill Thomson, Nicola Crossland, Fiona Dykes, Sharon McCann, Marion Campbell

**Affiliations:** 1 University of Aberdeen, Aberdeen, UK; 2 University of Stirling, Stirling, UK; 3 University of Central Lancashire, Lancashire, UK

## Background

It is recognised that patient and public involvement (PPI) in research should begin at the earliest stage possible. Whilst this ideal is widely acknowledged, in practice, it is not easily achievable. Constraints of time, funding, ethics and availability of appropriate representatives often mean that PPI is not included until research is fairly well advanced. This jeopardises one object of PPI; to ensure that research is being carried out **‘with'** or **‘by'** members of the public rather than **‘to**', **‘about**' or **‘for'** them, because it marginalises members of the public during the crucial project scoping and design phases.

## Methods

In a platform study to inform the design of trials delivering incentives for smoking cessation in pregnancy and breastfeeding, two service user groups, with similar characteristics to the intended target population, were co-applicants on the grant and worked closely with researchers based in Scotland and England throughout.

## Findings

The mother and baby groups contributed to interpreting systematic review findings, informed the text for participant materials, piloted study vignettes that were used within interview schedules, piloted the discrete choice experiment and voted on a shortlist of promising incentives.

## Conclusion

Extensive involvement of the groups as team members across a diverse range of research activities, from the study onset to dissemination, resulted in unique contributions to trial development. Regularly taking the research to the groups enabled harder to reach, less "professionalised" PPI involvement and the benefits of both group continuity and new members contributing.

